# Watching sport on television, physical activity, and risk of obesity in older adults

**DOI:** 10.1186/1471-2458-14-10

**Published:** 2014-01-08

**Authors:** Mark Hamer, Richard Weiler, Emmanuel Stamatakis

**Affiliations:** 1 Population Health Domain Physical Activity Research Group, Department of Epidemiology and Public Health, University College London, 1-19 Torrington Place, London WC1E 6BT, UK; 2 University College London Hospitals NHS Foundation Trust, London, UK; 3 Prevention Research Collaboration, School of Public Health, University of Sydney, Sydney, Australia

**Keywords:** Ageing, Epidemiology, Health promotion, Obesity, Physical activity, Sedentary

## Abstract

**Background:**

Television (TV) viewing has been associated with obesity although the effects of specific TV content on health and other behaviours remains unknown. We examined the association between watching sport on TV, physical activity levels, and risk of obesity.

**Methods:**

We studied 6,733 (aged 64.9 ± 9.2 yrs) men and women from the English Longitudinal Study of Ageing, a prospective study of community dwelling older adults. Data were collected on self reported TV time and content, and physical activity. Nurses measured height and weight for the calculation of body mass index.

**Results:**

On average, participants reported viewing TV for 5.3 ± 4.1 hours per day and 30.3% of the sample watched sport on TV at least twice a week. There was no association between watching sport and physical activity levels. Participants that watched sports every day were at higher risk of obesity [odds ratio = 1.39, 95% CI, 1.15, 1.68) after adjustment for age, sex, smoking, alcohol, physical activity, total TV time, disability, and self-rated health.

**Conclusions:**

Watching elite athletes may have no role in the promotion of physical activity in older adults, which has implications for staging large sporting events with physical activity legacy promises.

## Background

Excessive amounts of sedentary behaviours, such as viewing television (TV) , have been linked to a range of metabolic health risks [1-4], and TV time has been associated with BMI and waist circumference in older adults indepedently of physical activity participation [5]. Watching sport, whether on TV or live at a venue, predominantly involves a prolonged sedentary period, which has become more widespread with the introduction of all-seater stadiums in some sports [6]. Whilst the effects of specific TV content on behaviours remains unknown, food and beverage advertising are commonplace at major sporting events [7], which have been linked to eating habits and obesity [8]. It has, however, been suggested that watching sporting events, such as the Olympics, may inspire individuals to take up sports, become more physically active and justify staging of such events with physical activity legacy promises [9]. The aim of this study was to examine the association between watching sport on TV, physical activity levels, and risk of obesity in a representative sample of older adults from the UK.

## Methods

### Participants and study design

We studied 6,733 (aged 64.9 ± 9.2 yrs) men and women from the English Longitudinal Study of Ageing (ELSA), an ongoing cohort study that contains a nationally representative sample of the English population living in households [10]. The first wave of data was collected in 2002–03, although for the present study we used data collected at wave 4 (2008–09) when information on TV viewing was first gathered. Participants gave full informed written consent to participate in the study and ethical approval was obtained from the London Multi-centre Research Ethics Committee.

### TV viewing and physical activity

Participants were asked to recall “How many hours of television do you watch on an ordinary day or evening, that is, Monday to Friday?” and “How many hours of television do you normally watch in total over the weekend, that is, Saturday and Sunday?” Average daily time spent watching TV was calculated as {(weekday TV time x 5) + (Weekend TV time)}/7. Participants were also asked questions regarding TV content that was categorised as the frequency per week that they watched specific types of programmes : ‘How often do you watch sports on TV’.

We have described the ELSA physical activity measurements in detail previously [11]. In brief, participants were asked how often they took part in three different types of physical activity: vigorous, moderate- and low-intensity physical activity. Before answering, participants were shown prompt cards to help them interpret different PA intensities. Examples of moderate intensity activity included gardening, cleaning the car, walking at moderate pace, dancing, and floor or stretching exercises; vigorous intensity included running/jogging, swimming, cycling, aerobics/gym workout, tennis, and digging with a spade. The response options were: more than once a week, once a week, one to three times a month and hardly ever/never. Physical activity was further categorized into a binary variable based on reporting moderate or vigorous activity more than once a week.

### Obesity

Nurses collected anthropometric data (weight, height). Participants’ body weight was measured using Tanita electronic scales without shoes and in light clothing, and height was measured using a Stadiometer with the Frankfort plane in the horizontal position. Body mass index (BMI) was calculated using the standard formulae [weight (kilograms)/height (meters) squared]. Obesity was defined as BMI ≥ 30 kg/m^2^.

### Covariates

Demographic and health-related questions included cigarette smoking (current, previous or non-smoker), frequency of alcohol intake (daily, 5-6/wk, 3-4/wk, 1-2/wk, 1-2/month, once every couple of months, 1-2/year, never), self rated health (excellent, very good, good, fair, poor) and disability. We assessed disability based on participants’ responses to questions on perceived difficulties in basic (e.g., difficulty dressing, including putting on shoes and socks) [12] and instrumental (e.g., difficulty preparing a hot meal) activities of daily living (ADL) [13]. Participants with difficulties in one or more activities were considered to have some degree of disability.

### Statistical analysis

We used χ2 (for categorical) and ANOVA tests (for continuous data) to examine differences in characteristics across sports TV viewing groups. We calculated odds ratios (OR) and 95% confidence intervals (CI) for the risk of obesity in relation to viewing sport, which was categorized into three groups (once or less a week, twice or more a week, or daily). We adjusted the models for age, sex, cigarette smoking, alcohol intake, physical activity, self-rated health, disability, and total daily TV viewing (<2 hrs/d; 2 to <4 hrs/d; 4 to <6 hrs/d; ≥6 hrs/d). Modification by sex was explored by fitting a sex by sports viewing interaction term. In addition, we examined the association beween viewing sport on TV and physical activity using binary logistic regression with physical activity as the depedent variable (categorised into inactive or active based on participation of any moderate or vigorous activity more than once a week). All analyses were conducted using SPSS version 20.

## Results

On average, participants reported viewing TV for 5.3 ± 4.1 hours per day and 30.3% of the sample watched sport on TV at least twice a week. Participants that viewed sport were more likely to be men, reported greater total TV viewing time, impairments in ADL/IADLs and rate their health as poor (Table 1). 69.9% of the sample reported participating in moderate or vigorous activity more than once a week and 21.6% of the sample were vigorously active more than once a week. There was no association between watching sport and physical activity levels (p-trend = 0.19) ; in comparison with participants watching sport once or less a week, those that watched sport twice or more a week (OR =1.15, 95% CI, 0.98 – 1.34), or daily (OR =1.01, 95% CI, 0.83 – 1.24) were no more likely to be active, after adjusting for age, sex, smoking, alcohol, total daily TV time, impairments in ADLs/IADLs, and self rated health. Similarly, when we used only vigorous activity as the dependent variable there was no association between watching sport and participation in vigorous activity (p-trend = 0.08).

**Table 1:** Characteristics of the sample in relation to viewing sport on television

**Variable**	** Viewing sport on TV**			**p-value**
	**Once or less a week**	**Twice or more a week**	**Daily**	
Mean age (yrs)	64.5 ± 9.3	65.7 ± 9.1	65.7 ± 9.0	<0.001
Sex (% men)	36.3	68.9	77.0	<0.001
Smoking (%)	12.7	9.8	13.4	0.007
Alcohol (% at least 5/wk)	22.7	27.9	23.3	<0.001
Mean total TV viewing (hrs/d)	5.0 ± 4.0	5.6 ± 3.9	6.8 ± 4.9	<0.001
Physical activity (% active)^†^	69.2	73.6	66.6	0.001
Impairements in ADL/IADLs	23.2	20.5	26.4	0.001
Self rated health (% poor)	5.1	4.3	7.7	0.001
Mean body mass index (kg/m^2^)	28.0 ± 5.3	28.5 ± 5.0	28.9 ± 5.3	<0.001

^†^defined as moderate or vigorous activity more than once a week.

Obesity was prevalent in 30.8% of the sample. Watching sport on TV was associated with higher risk of obesity in a dose–response manner (Table 2), and these associations persisted after adjustment for covariates, including total TV viewing time and physical activity. The sex interaction term was significant (p = 0.026), and sex stratified analysis showed that associations between daily sports viewing and obesity were evident in men (multivariable adjusted OR = 1.57, 95% CI, 1.24 – 1.97) but not women (OR = 1.08, 95% CI, 0.75 – 1.55). For each 2 hr/d increase in total TV viewing there was an incremental increase in obesity risk (OR = 1.28, 95% CI, 1.21 – 1.36) after adjusting for age, sex, smoking, alcohol, physical activity, impairments in ADLs/IADLs, and self-rated health.

**Table 2:** Watching sports on TV and risk of obesity in older adults (n = 6,733)

**Frequency of viewing sport on TV**	**Cases/N**	**Model 1**	**Model 2**
		**OR (95% CI)**	**OR (95% CI)**
Once or less a week	1387/4690	Reference	Reference
Twice or more a week	443/1410	1.21 (1.06, 1.38)	1.17 (1.01, 1.35)
Daily	242/633	1.67 (1.40, 2.00)	1.39 (1.15, 1.68)
P-trend		<0.001	0.001

Model 1 adjusted for age and sex.

Model 2 adjusted for age, sex, physical activity, smoking, alcohol, total daily TV time, impairments in ADLs/IADLs, self rated health.

## Discussion

The main aim of this study was to examine the association between viewing sport on TV with physical activity levels and obesity. It is likely that greater numbers of people watch sport on TV than live at the venue and viewing TV is therefore a greater public health concern. Contrary to the assumption that watching sport on TV may be associated with greater uptake of physical activity, we found no evidence to support this hypothesis. In fact, more frequent watchers of sport on TV demonstrated the highest amount of daily time spent watching TV and a higher risk of being obese. The association between viewing sports on TV and obesity was particularly evident in men.

These findings have relevance for the promotion of physical activity to the general population, especially in light of current debate surrounding the justification for holding Olympic and major sporting events and health legacy pledges [14]. Although higher than average levels of sports participation were recorded in the late summer period of 2012, immediately after the Olympic Games, more recent data released from Sport England [15] suggests negligible differences in sports participation compared with the same period prior to the games. This is largely consistent with other evidence examining the impact of major sporting events on population physical activity levels and health [16,17]. Evidence suggests that uptake of physical activity is optimised in the presence of peer support or when peers are used as role models [18,19]. However, if the social norm and our true peer group are watching sport on TV, rather than participating in sport, then lack of evident behavioural effect is completely understandable. Using peers as role models is also known to increase self efficacy, a key driver in the uptake of physical activity. Watching elite athletes may therefore have no role in the promotion of physical activity in older adults.

The association between viewing sport and obesity was independent of total TV viewing time, suggesting that other mechanisms might also partly explain the effects. One possibility is that participants consume more unhealthy foods and beverages whilst watching sports, which may in part be driven by sponsorship relationships between sporting organisations and food and beverage brands [7]. Indeed, experimental evidence has demonstrated that food advertising increased consumption of products not in the presented advertisements, and these effects were not related to reported hunger or other conscious influences [8]. Nevertheless, we were unable to adjust for dietary intake in our analysis. Our data are based on self-report that might have introduced bias, thus we cannot discount the possibility of residual confounding. We have, however, previously demonstrated the validity of measures such as self-reported morbidity in ELSA [20]. Physical activity was assessed crudely although the measure has demonstrated excellent convergent validity in grading a plethora of psychosocial, physical and biochemical risk factors [11,21]. Since these analyses were cross-sectional we cannot discount the possibility of reverse causation in that obesity and poorer general health causes people to spend more time sedentary and not the reverse. Indeed, a recent study in middle-aged and older adults demonstrated that BMI at baseline was prospectively associated with greater TV viewing at follow-up but not the converse [22]. Lastly, this study was restricted to older adults and we do not know if the same null associations between watching sport on TV and physical activity would be observed in younger adults who might more readily identify with their sport playing peer group.

## Conclusions

In conclusion, we found no association between watching sport on TV and physical activity levels in a representative sample of older adults. These data question using health legacy pledges as a vehicle to justify staging major sporting events.

## Abbreviations

TV: Television; ELSA: English Longitudinal Study of Ageing; BMI: Body mass index; OR: Odds ratio; IADL: Instrumental activities of daily living; ADL: Activities of daily living.

## Competing interests

None of the authors have any competing interests to declare.

## Authors’ contributions

MH had full access to the data, and takes responsibility for the integrity and accuracy of the results. MH drafted the paper. All authors contributed to the concept and design of study, drafting and critical revision of the manuscript. All authors read and approved the final manuscript.

## Pre-publication history

The pre-publication history for this paper can be accessed here:

http://www.biomedcentral.com/1471-2458/14/10/prepub
